# Accuracy of oxidative stress markers for predicting delayed graft function lasting longer than a week in deceased-donor kidney transplantation

**DOI:** 10.31744/einstein_journal/2026AO1827

**Published:** 2026-04-07

**Authors:** João Paulo Ribeiro, Thales Paulo Batista, Cristiano Souza Leão, Leuridan Cavalcante Torres, Danielle da Silva Dias, Kátia De Angelis

**Affiliations:** 1 Instituto de Medicina Integral Professor Fernando Figueira Recife PE Brazil Organ Procurement Organization, Instituto de Medicina Integral Professor Fernando Figueira, Recife, PE, Brazil.; 2 Hospital da Restauração Department of Surgery Recife PE Brazil Department of Surgery, Hospital da Restauração, Recife, PE, Brazil.; 3 Universidade Federal de São Paulo Division of Translational Medicine São Paulo SP Brazil Division of Translational Medicine, Universidade Federal de São Paulo, São Paulo, SP, Brazil.; 4 Universidade Federal de Pernambuco Department of Surgery Recife PE Brazil Department of Surgery, Universidade Federal de Pernambuco, Recife, PE, Brazil.; 5 Instituto de Medicina Integral Professor Fernando Figueira Department of Surgery Recife PE Brazil Department of Surgery, Instituto de Medicina Integral Professor Fernando Figueira, Recife, PE, Brazil.; 6 Instituto de Medicina Integral Professor Fernando Figueira Recife PE Brazil Translational Research Laboratory Prof. CA Hart, Instituto de Medicina Integral Professor Fernando Figueira, Recife, PE, Brazil.; 7 Universidade Federal de São Paulo Department of Physiology São Paulo SP Brazil Department of Physiology, Universidade Federal de São Paulo, São Paulo, SP, Brazil.

**Keywords:** Kidney transplantation, Oxidative stress, Graft survival

## Abstract

**Objective:**

To assess the accuracy of oxidative stress markers in predicting delayed graft function (DGF) lasting more than one week after deceased-donor kidney transplantation (KTx).

**Methods:**

A translational pilot study was carried out and a five-step hierarchical diagnostic accuracy strategy was applied to evaluate the predictive value of oxidative stress markers for DGF >7 days in adult patients with end-stage kidney disease undergoing deceased-donor KTx. Statistics comprise multiple conventional methods, C-statistics, and diagnostic test performance measures.

**Results:**

Cohort analysis of 27 consecutive patients revealed that 33.3% (9/27) developed DGF >7 days. Assessment of the oxidative stress markers selected in the first statistical step comparing donors and controls revealed a significant correlation of the DGF duration with the recipient's protein oxidation (ρ=0.466; p=0.022) as well as donor's hydrogen peroxide (ρ=-0.489; p<0.014). The areas under the curve were 0.683 (95% confidence interval [95%CI], 0.458-0.859; p=0.224) and 0.746 (95%CI=0.534-0.897; p=0.019), respectively. A cutoff of ≤14 *μ*M H_2_O_2_ had the highest level of discriminating power for predicting DGF >7 days. The sensitivity, specificity, positive predictive value, negative predictive value, and overall diagnostic accuracy to identify patients at risk for experiencing DGF >7 days was 85.7%, 61.1%, 46.1%, 91.7%, and 68%, respectively.

**Conclusion:**

Donor hydrogen peroxide was identified as a potential predictor of DGF >7 days in patients who underwent deceased-donor KTx. A cut-off value of ≤14 *μ*mol/L is suggested to stratify patients at high risk of requiring dialysis beyond the first post-transplant week.

## In Brief

The role of oxidative stress markers in predicting clinical outcomes of deceased-donor kidney transplantation remains unclear. In this prospective pilot study, Ribeiro Neto et al. identified donor serum hydrogen peroxide as a potential predictor of delayed graft function lasting >7 days.

## Highlights

■ Oxidative stress markers are associated with the intensity and consequences of ischemia-reperfusion injury during deceased-donor kidney transplantation.
■ The donor's serum hydrogen peroxide level achieved 85.7% sensitivity and a 91.7% negative predictive value for detecting delayed graft function lasting more than one week.
■ Donor H_2_O_2_ ≤14*μ*M is suggested to dichotomize risk for delayed graft function lasting longer than a week.
■ This study provides pivotal information on the use of oxidative stress markers to identify patients at high risk of requiring dialysis beyond the first post-transplant week.

## Graphical abstract

**Figure:**
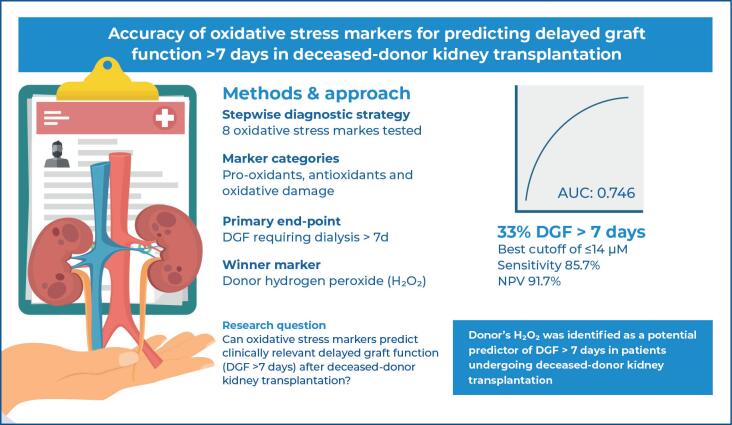

## INTRODUCTION

Ischemia-reperfusion is an inherent phenomenon in solid organ transplantation that leads to a tonic increase in oxidative stress markers during kidney transplantation (KTx).^(1)^ These biochemical indicators reflect the balance between cellular oxidative injury and the homeostatic ability of the body to detoxify reactive species or repair resulting cellular damage.^(2,3)^ Notably, oxidative stress markers have been associated with relevant complications following KTx^(2,3)^ and may serve as valuable predictors of clinically significant outcomes after deceased-donor KTx.^(1,2)^ Nevertheless, their role in predicting clinical outcomes remains unclear and warrants further investigation.

Kidney transplantation remains the therapy of choice for improving outcomes in patients with end-stage kidney disease, with benefits increasing over time despite the increasing age and comorbidity burden among contemporary transplant recipients.^(4–6)^ Unfortunately, renal graft recovery remains a critical issue after KTx, with many patients experiencing delayed graft function (DGF). This ultimately imposes a significant financial burden because of prolonged hospitalization and the need for additional dialysis. Additionally, allograft dysfunction strongly influences the long-term outcomes after deceased-donor KTx. The incidence of allograft dysfunction ranges from 20-30% in the United States^(7)^ to nearly 50% in Europe,^(8)^ and reaches rates of 54-62% in Brazilian centers.^(9,10)^

Considering the well-established impact of DGF on graft survival and patient outcomes,^(10–12)^ we hypothesized that oxidative stress markers could serve as valuable predictors of DGF >7 days following deceased-donor KTx,^(1,2)^ thereby providing a potential tool for improving patient selection, enabling early intervention, and optimizing post-transplant management.

## OBJECTIVE

To assess the accuracy of oxidative stress markers for predicting DGF >7 days in patients who have undergone deceased-donor kidney transplantation.

## METHODS

### Study design

A translational pilot study was conducted, and a stepwise diagnostic accuracy strategy was applied to assess the predictive value of oxidative stress markers for DGF >7 days in adult patients with chronic end-stage kidney disease who underwent deceased-donor KTx. Following the Standards for Reporting of Diagnostic Accuracy Studies (STARD) Statement (https://www.equator-network.org/reporting-guidelines/stard/), we prospectively explored systemic levels of oxidative stress damage by thiobarbituric acid reactive substances (TBARS) and carbonyls; antioxidants by superoxide dismutase (SOD), catalase (CAT) activities and ferric reducing antioxidant power (FRAP); and pro-oxidants by hydrogen peroxide, nicotinamide adenine dinucleotide phosphate (NADPH) oxidase, and nitrite, in both recipients and donors, as predictors of DGF >days. All patients underwent the operation at the *Instituto de Medicina Integral Professor Fernando Figueira* (IMIP) between June 2021 and June 2022. This study was reviewed by the Ethics Research Committee of the IMIP (CAAE 42858520.8.0000.5201, # 4.556.284) and complied with the current Brazilian ethical guidelines.

### Study population and procedures

In terms of the scope, we limited our study to consecutive deceased-donor KTx performed in adult patients (≥18 years) with chronic end-stage kidney disease during the 1-year period of laboratory assessment that we defined at our convenience (1-year cohort study). Additional requirements for inclusion were transplantation being carried out at IMIP, with kidney graft retrieval performed at the *Hospital da Restauração* (RH), and voluntary informed consent provided by the recipients and donors’ families for participation in this study. The only exclusion criterion was missing data from patient charts. All kidney transplantations were performed by the same surgical team following standardized institutional protocols and routines.^(13)^ During the study period, the patients received the same institutional standards of immunosuppressive therapy, including induction with an anti-interleukin-2 receptor antibody, calcineurin inhibitor, mycophenolate, and steroids.

### Blood sampling and data collection

As part of our prospective study, we assessed and recorded clinical and paired laboratory data using electronic spreadsheets. Blood samples for oxidative stress marker analysis were collected at standardized time points to ensure procedural consistency. From the donors, peripheral blood was drawn immediately prior to the initiation of procurement surgery, and a sample of the preservation solution was obtained at the time of organ perfusion. In recipients, the first peripheral blood sample was collected upon hospital admission, before the administration of immunosuppressive therapy. A second sample was drawn from the renal vein immediately after graft reperfusion. All procedures were conducted under the direct supervision of the research team to ensure adherence to the protocol. Blood samples were processed using standard methods^(14)^ as detailed in the Table 1S, Supplementary Material. The endpoint of clinical interest was the severity of DGF, assessed as DGF >7 days, in patients requiring dialytic treatment beyond the first week after KTx.^(10–12)^

### Statistical analysis

The predictive value of the oxidative stress markers was assessed using a five-step hierarchical strategy. First, donor and recipient results were compared with those of healthy controls. Second, statistically significant parameters were correlated with the DGF duration using Spearman's rank test. Third, the overall accuracy was evaluated using the C-statistic, and optimal cut-offs were determined using Youden's J index. Fourth, significant continuous variables (i.e., area under the curve ≥0.7 and p<0.05) were dichotomized and reassessed using the χ^2^ test. Finally, the diagnostic accuracy was determined by calculating the sensitivity, specificity, positive predictive value, negative predictive value, and overall accuracy.

Descriptive statistics are summarized as median (interquartile range) or frequency (percentage). Between-group comparisons were performed using the Mann-Whitney U test or χ^2^ test, including Yates's correction or Fischer's exact test as appropriate. Analyses were performed using MedCalc v.19.4 (MedCalc Software, Ostend, Belgium), and correlation strength was classified according to ρ-values as very strong (0.91-1), strong (0.71-0.9), moderate (0.51-0.7), weak (0.31-0.5), very weak (0.01-0.3), and no correlation (zero), as previously reported.^(15)^ P-values (two-tailed) <0.05 were considered to indicate a statistically significant difference.

## RESULTS

Our cohort analysis included 27 recipients and 20 donors who consented to participate in our study. The control group comprised 19 healthy participants with a median age of 43 years (interquartile range [IQR] 35.5-46) and a male/female ratio of 1.7, which we obtained from our laboratory database of oxidative stress markers. The baseline demographics of the main donors and recipients are shown in table 1. In terms of renal function recovery, the median DGF duration was 4 days (IQR, 1-14 days), with DGF occurring in 77.8% (n=21/27) of the patients. Delayed recovery requiring dialytic treatment beyond the first week after transplantation was observed in 33.3% (9/27) of cases, and prolonged DGF lasting more than 14 days was found in 25.9% (n=7/27). Most patients (92.6%; n=25/27) achieved resolution of DGF within 28 days of receiving a deceased-donor kidney graft.

**Table 1:** Baseline demographic characteristics

Characteristics		n (%) or median (IQR)
Recipient factors		
Age (years)		46 (36.5-59)
Sex		
	Male	15 (55.6)
	Female	12 (44.4)
Body mass index (kg/m^2^)		24.4 (22.6-25.8)
Time on dialysis (months)		78 (48-105)
Comorbidities		
	Hypertension	19 (70.4)
	Diabetes mellitus	2 (7.4)
End-stage kidney disease		
	Mixed/undefined	16 (59.3)
	Chronic obstruction/infection	5 (18.5)
	Others	6 (22.2)
Donor factors		
Age (years)		39 (24-53.5)
Sex		
	Male	20 (74)
	Female	7 (26)
Cause of death		
	Trauma	12 (44.4)
	Stroke	12 (44.4)
	Others	3 (11.2)
Expanded criteria donor		4 (14.8)
HLA-PRA		
	0%	21 (77.8)
	1-79%	5 (18.5)
	≥80%	1 (3.7)
Cold ischemia time (hours)		22.5 (17-29.5)
Body mass index (kg/m^2^)		25.9 (25.1-29.3)
Creatinine at transplantation (mg/dL)		1.3 (1.1-1.9)
Kidney donor risk index		0.86 (0.68-1.42)
Kidney donor profile index		35 (13-83)

HLA: human leukocyte antigen.

**Figure 1:**
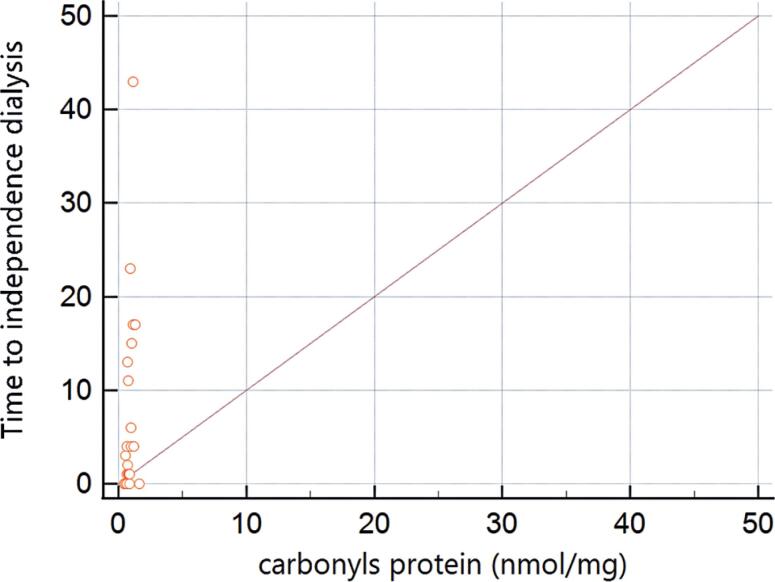
Scatterplot of the correlation between carbonyls and the duration of delayed graft function

**Figure 2:**
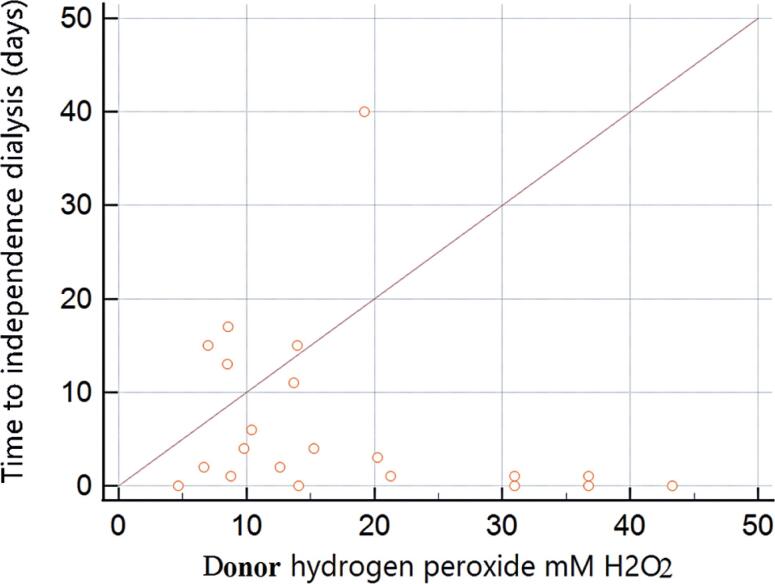
Scatterplot of the correlation between hydrogen peroxide and the duration of delayed graft function

Table 2 summarizes the systemic (blood) levels of oxidative stress markers. Compared with controls, there was no significant difference in catalase levels in the recipient (p=0.146) or donor (p=0.351) samples. The same was true for TBARS (p=0.914) and NADPH oxidase (p=0.690) in recipient samples, and for nitrite (p=0.260) in donor samples. Assessment of the correlation between the remaining markers with the DGF duration reached statistical significance for the recipient's protein oxidation by carbonyls (ρ=0.466; p=0.022) and the donor's hydrogen peroxide (ρ=-0.489; p<0.014). A summary of the correlations between all oxidative stress markers and the DGF duration is shown in table 3, and graphical assessments of the correlations for the oxidative stress markers selected in this step are presented in figures 1 and 2.

**Table 2:** Summary of the systemic (blood) oxidative stress markers

Variables	Median (IQR)			p values#	
	Controls (A)	Donors (B)	Recipients (C)	A *versus* B	A *versus* C
TBARS	6.20 (5.45-7.28)	7.32 (6.71-8.50)	6.20 (5.80-6.98)	0.027	0.914
Carbonyls (nmol/mg)	1.43 (1.32-1.46)	0.67 (0.52-0.80)	0.86 (0.68-0.98)	<0.001	< 0.001
SOD	5.36 (5.21-5.55)	7.18 (6.11-9.19)	6.81 (6.22-7.96)	<0.001	< 0.001
CAT	2.10 (1.68-4.42)	2.45 (1.49-3.19)	1.99 (1.35-2.53)	0.351	0.146
FRAP	1.62 (1.07-1.73)	2.89 (1.64-5.31)	3.43 (2.39-5.16)	0.024	< 0.001
Hydrogen Peroxide (*μ*M H_2_O_2_)	5.15(2.42-7.73)	13.96 (8.74-21.20)	28.80 (18.70-50.20)	<0.001	< 0.001
NADPH oxidase	32 (25-44.90)	17.60 (12.40 −33.10)	34 (26.80-44.60)	<0.001	0.690
Nitrite (nmol/mg of protein)	0.40 (0.32-0.66)	0.57 (0.38-1.18)	0.67 (0.52-1.09)	0.260	0.006

# Mann-Whitney U-test between donors and recipients versus controls.

TBARS: thiobarbituric acid reactive substances (pmoles/mg); SOD: superoxide dismutase (Usod/mg); CAT: catalase (nmol/mg); FRAP: ferric reducing antioxidant power (mM Fe [II]); NADPH oxidase: nicotinamide adenine dinucleotide phosphate oxidase (nmol/mg of protein).

**Table 3:** Summary of correlations between oxidative stress markers and the DGF duration

Laboratorial marker		Spearman's correlation statistics		
		ρ values (rho)	p value	Strength of the correlation
Recipient marker				
	Carbonyls (nmol/mg protein)	0.464	0.022	Weak
	SOD	-0.155	0.449	Very weak
	FRAP	0.018	0.943	Very weak
	Hydrogen Peroxide (*μ*M H_2_O_2_)	-0.013	0.956	Very weak
	Nitrite (nmol/mg protein)	0.019	0.937	Very weak
Donor Marker				
	Carbonyls (nmol/mg protein)	0.050	0.982	Very weak
	TBARS	0.011	0.956	Very weak
	SOD	0.091	0.667	Very weak
	FRAP	0.036	0.860	Very weak
	NADPH oxidase	-0.313	0.136	Very weak
	Hydrogen peroxide (*μ*M H_2_O_2_)	-0.490	0.014	Weak

SOD: superoxide dismutase (Usod/mg); FRAP: ferric reducing antioxidant power (mM Fe(ii]); TBARS: thiobarbituric acid reactive substances (pmoles/mg); CAT: catalase (nmol/mg); NADPH oxidase: nicotinamide adenine dinucleotide phosphate oxidase (nmol/mg of protein).

**Figure 3:**
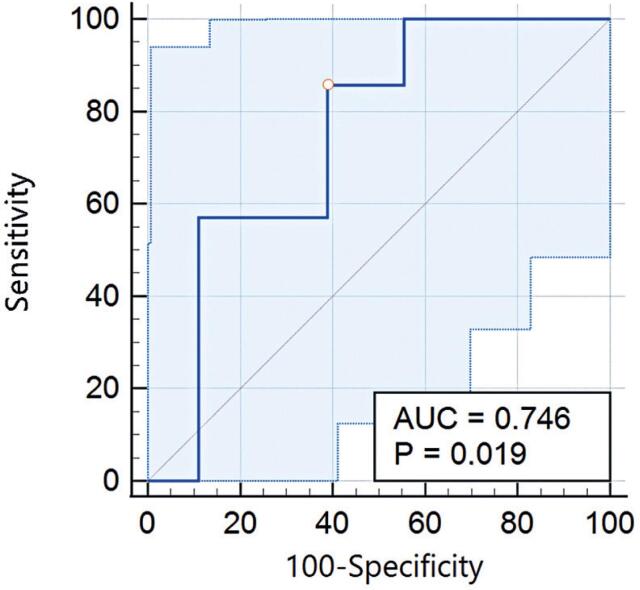
Receiver operating characteristic curve for the overall discriminatory power of the donor's hydrogen peroxide level to predict DGF >7 days. AUC, area under the receiving operating characteristic curve

According to the C-statistic, the overall accuracy of recipients’ protein oxidation by carbonyls and donors’ blood hydrogen peroxide levels (as a continuous variable) for predicting DGF >7 days was 0.683 (95% confidence interval [95%CI]=0.458-0.859; p=0.224) and 0.746 (95%CI=0.534-0.897; p=0.019), respectively. A graphical summary of the receiver operating characteristic curve for the donor hydrogen peroxide is presented in figure 3. A cutoff of ≤13.963 *μ*M H_2_O_2_ was found to have the highest discriminating power for predicting the DGF >7 days, reaching a Youden's J index of 0.468. The level of hydrogen peroxide in the donor's blood dichotomized by the rounded cutoff (*i.e*.: ≤14 *μ*M H_2_O_2_ as a positive test to identify high-risk patients for experiencing DGF >7 days) was also confirmed to be associated with DGF >7 days according to the Fisher's exact test results (p=0.039).

Among the seven donor-recipient pairs analyzed, two pairs presented discordant outcomes, with one recipient developing DGF >7 days and the other not. In these cases, one recipient tested positive (hydrogen peroxide ≤14 *μ*M H_2_O_2_), while the other tested negative (hydrogen peroxide >14 *μ*M H_2_O_2_). Additionally, in one concordant pair where both recipients developed DGF >7, the level of hydrogen peroxide was >14 *μ*M H_2_O_2_. Overall, the sensitivity, specificity, positive predictive value, negative predictive value, and accuracy of donor's hydrogen peroxide levels, when dichotomized as a positive or negative test, were found to be 85.7% (95%CI=42.1-99.6), 61.1% (95%CI=35.7-82.7), 46.1% (95%CI=30.8-62.2), 91.7% (95%CI=63.2-98.6), and 68% (95%CI=45.6-85.1), respectively.

## DISCUSSION

### Summary of the main results

In this pilot study, we explored the value of eight oxidative stress markers in both recipient and donor blood samples to predict clinically relevant endpoints in patients who underwent deceased-donor kidney transplantation. Accordingly, we identified the systemic level of donor's hydrogen peroxide as a potential predictor of DGF >7 days by applying a comprehensive stepwise diagnostic approach. Herein, the donor systemic levels of this oxidative stress marker were found to be higher than those in healthy controls and correlated with the DGF duration after transplantation. Using c-statistics, we also determined the optimal cutoff for dichotomizing this marker as a positive/negative test. The cutoff was then statistically associated with DGF >7 days, achieving 85.7% sensitivity and a 91.7% negative predictive value.

### Results in the context of published literature

Clinical factors such as the donor and recipient age, ischemia time, and revision surgery in association with human leukocyte antigen mismatching have been the most explored predictors for the occurrence of DGF in patients undergoing KTx.^(16–19)^ On the other hand, markers of oxidative stress have also been demonstrated a potential value in a few numbers of studies. For example, by exploring the role of malondialdehyde as an early predictive marker of graft dysfunction, Fonseca et al.^(2)^ found that the levels of this product of lipid peroxidation were significantly higher shortly after KTx and increased further in the subsequent days in patients developing DGF. In these settings, the authors reported that malondialdehyde levels accurately predicted DGF and demonstrated that high levels at one week were independently associated with poorer 1-year allograft function.^(2)^ Similarly, assessing the oxidative status by the OxyScore and AntioxyScore indexes, Rodriguez-Sanchez et al.^(3)^ recently demonstrated that donation after circulatory death induced greater short-term oxidative, whereas the early levels of oxidative damage were predictive of the graft function at 1 year among recipients of donation after brain death. Among the eight oxidative stress markers explored in recipient and donor blood samples, we added the donor's hydrogen peroxide level at the time of KTx as a potential predictor of DGF >7 days.

Because of a long cold ischemia period and other socioeconomic-related factors, the rates of DGF have remained high in Brazil, ranging from 29.9% to 87.7% among different transplantation centers.^(9)^ Accordingly, we observed a DFG rate of 77.8% in the current study despite a lower median DGF duration (4 days) in comparison with the American (10 days)^(12)^ and European (5 days) series.^(11)^ Of note, the length of the DGF, rather than the DGF itself, is associated with long-term kidney graft function according to several studies.^(10–12,20)^ As confirmation of the duration-dependent effect of DGF on graft survival, the need for dialytic treatment beyond the first week after transplantation occurred in 33.3% of our cases and severe DGF lasting more than 14 days occurred in 25.9% of our cases, similar to 22.5%^(12)^ and 26%^(11)^ reported in large previous studies. Since most of our cases (92.6%) achieved resolution of DGF within 4 weeks after KTx at a rate similar to those previously reported,^(11, 12)^ we chose DGF >7 days as a more clinically relevant endpoint in our study.

### Strengths and weaknesses

The main strengths of this study include its comprehensive accuracy analysis using five stepwise approaches and the use of paired prospectively collected data to assess oxidative stress markers in both donor and recipient blood samples. To our knowledge, this study is the first to investigate oxidative stress markers as predictors of DGF in deceased-donor KTx. Notably, the study was conducted during the COVID-19 pandemic, which required additional logistical efforts to perform complex surgical procedures such as kidney transplantation and may have contributed to longer cold ischemia times and higher rates of DGF in this study. Although the number of procedures may seem limited, this is inherent to the pilot study design, and reflects the rigorous methodological control applied to sample collection at each stage of the donation and transplantation processes.

### Implications for practice and future research

Oxidative stress, one of the most important components of the ischemia-reperfusion process, has been implicated in relevant complications after KT, including DGF and allograft rejection.^(2, 3)^ However, effective treatment of oxidative stress in transplant patients requires further investigation.^(21)^ Additionally, identifying patients at high risk of allograft malfunction is important to improve KTx outcomes and graft allocation policies. Overall, our pilot study provides pivotal information that will aid researchers in exploring oxidative stress markers in future large clinical studies. These studies should focus on identifying patients who require early intervention and intensive postoperative surveillance to mitigate the effects of ischemic injury and DGF on long-term graft function.

## CONCLUSION

We identified the donor's hydrogen peroxide as a potential predictor of DGF >7 days in adult patients with end-stage kidney disease who underwent deceased-donor KTx. This oxidative stress marker warrants further investigation in larger clinical studies, with a suggested cut-off value of ≤14*μ*M H_2_O_2_ to identify patients at risk of requiring dialysis beyond the first week post-transplant.

## Data Availability

We have no plan to make individual participant data available to other researchers because data sharing was not required in the study protocol, which was initially reviewed and approved by our Ethics Research Committee.
