# Supplementary material for: Accuracy of oxidative stress markers for predicting delayed graft function lasting longer than a week in deceased-donor kidney transplantation
Source: Einstein (Sao Paulo). 2026 Apr 7;24:eAO1827. doi: 10.31744/einstein_journal/2026AO1827 (PMC13128223; doi:10.31744/einstein_journal/2026AO1827)
Supplement: SUPPLEMENTARY MATERIAL [file 2317-6385-eins-24-eAO1827-suppl01.pdf]

SUPPLEMENTARY MATERIAL

Accuracy of oxidative stress markers for predicting delayed graft function lasting longer than a week in deceased-donor kidney transplantation

João Paulo Ribeiro Neto, Thales Paulo Batista, Cristiano Souza Leão, Leuridan Cavalcante Torres, Danielle da Silva Dias, Kátia De Angelis

DOI: 10.31744/einstein_journal/2026AO1827

Table 1S. Oxidative stress assessment in blood samples

Preparation of plasma, serum, and erythrocytes

Plasma (EDTA tube) and serum (dry tube) were centrifuged for 20 min at 3000rpm in a refrigerated centrifuge between 0 and 4°C (Eppendorf, 5804-R), and the supernatant was frozen in a freezer at -80°C for oxidative stress and inflammation measurements. Additionally, the erythrocytes were washed three times with saline and stored in a preservative solution ($MgSO_4$ and glacial acetic acid).

Protein Assay

Proteins were quantified by standard methods using a bovine albumin solution at a concentration of 1 mg/mL.

Protein Oxidation by Carbonyls (Protein Damage)

The carbonyl assay was used to detect oxidatively modified proteins. The technique is based on the reaction of oxidized proteins from blood plasma with 2,4 dinitrophenylhydrazine (DNPH) in an acidic medium, followed by successive washes with acids and organic solvents, and a final incubation with guanidine.

Thus, the absorbance of the carbonyls was measured in plasma using a spectrophotometer at 360nm in a reaction medium containing the following reagents: guanidine (6 M) in hydrochloric acid (HCl) (2.5M) at a pH of 2.5 or 2.4, DNPH in HCl (2.5M), trichloroacetic acid (TCA) 20%, TCA 10%, and ethanol-ethyl acetate 1:1 (V/V). In parallel, a protein standard curve was obtained using albumin, and the absorbance was read at 280nm using a spectrophotometer.

Thiobarbituric Acid-Reactive Substances (TBARS)

For the reaction to occur, 0.75mL of 10% (W/V) trichloroacetic acid (TCA) was added to 0.25mL of plasma, with the function of denaturing the proteins present and acidifying the reaction medium. This mixture was shaken and centrifuged for 3 min at 1000rpm. Next, 0.5mL of the supernatant was removed and 0.5mL of 0.67% (w/v) thiobarbituric acid (TBA) was added, which reacted with the lipoperoxidation products to form a pink compound. The mixture was incubated for 15 min at 100°C and then cooled on ice. Absorbance was read at 535nm using a spectrophotometer.

Catalase (CAT)

The rate of hydrogen peroxide decomposition is directly proportional to CAT activity. Thus, H_2O_2 consumption can be used as a measure of CAT activity. The test consisted of measuring the decrease in absorbance at 240nm, the wavelength where there is the greatest absorption by hydrogen peroxide, using quartz cuvettes. A phosphate buffer solution (50 mmol/L, pH 7.4) was used to perform the measurements. Next, 985μL of this buffer and 5μL the diluted erythrocyte sample were added to a spectrophotometer cuvette, and this mixture was discounted against a blank phosphate buffer. Lastly, 15μL of hydrogen peroxide (0.3 mol/L) was added. The decrease in absorbance was monitored using a spectrophotometer.

Superoxide Dismutase (SOD)

The SOD activity was determined based on the reaction of the superoxide radical with pyrogallol, with the formation of a colored product detected spectrophotometrically at 420nm for 2 min. The percentage inhibition of the initial reaction rates depended on the pH and the amount of SOD present in the reaction mixtures. The amount of enzyme required to inhibit the reaction by 50% was defined as one unit of SOD. The reaction mixture contained 980μL of 50mM tris-phosphate buffer pH 8.2, 10μL of 24mM pyrogallol, 5μL of 30mM CAT, and 5μL of sample. A standard curve with three different SOD concentrations (0.25, 0.5, and 1 U) was constructed to determine the equation used in the calculations.

Non-enzymatic Antioxidant Potential (FRAP)

The antioxidant assay for determining the reducing power of iron ions, FRAP (from the English Ferric Reducing Antioxidant Power), is based on the production of Fe^{2+} ions (ferrous form) by the reduction of Fe^{3+} ions (ferric form) present in the 2,4,6- tripyridyl -s- triazine (TPTZ) complex. Thus, the change in absorbance was directly related to the total reduction power of the antioxidant electron donation present in the reaction. When reduction occurred, the hue of the reaction mixture changed from light purple to intense purple. The higher the absorbance or intensity of the color, the greater the total antioxidant potential of the sample. The technique was performed in a microplate, in which 10μL of a standard solution of ferrous sulfate heptahydrate or 10μL of sample was added to 290μL of FRAP reagent (sodium acetate and acetic acid buffer, pH 3.6; 10mM TPTZ; 20mM ferric chloride hexahydrate). The microplate was then incubated for 5 min with shaking at 37ºC, and the absorbance was measured at 593nm.

NADPH Oxidase

NADPH oxidase activity was determined in erythrocytes and superoxide production was monitored using Enzyme-Linked Immunosorbent Assay (ELISA). To perform the assay, we used 50mM phosphate buffer containing 2mM EDTA and 150mM sucrose, 3mM NADPH1, and 10μL of sample.

Hydrogen Peroxide

Hydrogen peroxide was measured by the oxidation of phenol red mediated by horseradish peroxidase (HRP), which led to the formation of a measurable compound at 630nm. We performed a curve with distilled H_2O, H_2O_2 250μM, horseradish peroxidase solution (PRS) composed of buffer dextrose, phenol red (Sigma-Aldrich Corporation) horseradish peroxidase type II (Sigma-Aldrich Corporation), and sodium hydroxide (NaOH) (FMaia Gold). Then, 70μL of plasma was added to the ELISA plate together with 180μL PRS and incubated for 25 min at room temperature. After this period, we added 5μL of NaOH and readings were recorded using ELISA plate reader equipment.

Nitrite

Plasma nitrite levels (50μL) were measured by reacting samples with Griess reagent on microplates (96 wells) in an ELISA reader. The total tissue nitrite was estimated using a standard absorbance curve at 592nm.
